# Large spot transpupillary thermotherapy: A quicker laser for treatment of high risk prethreshold retinopathy of prematurity - A randomized study

**DOI:** 10.4103/0301-4738.77046

**Published:** 2011

**Authors:** Parag K Shah, V Narendran, N Kalpana

**Affiliations:** Department of Pediatric Retina and Ocular Oncology, Aravind Eye Hospital & Postgraduate Institute of Ophthalmology, Coimbatore, Tamil Nadu, India

**Keywords:** Large spot, laser treatment, retinopathy of prematurity, transpupillary thermotherapy

## Abstract

To compare structural and functional outcome and time efficiency between standard spot sized conventional pulsed mode diode laser and continuous mode large spot transpupillary thermotherapy (LS TTT) for treatment of high risk prethreshold retinopathy of prematurity (ROP). Ten eyes of five preterm babies having bilateral symmetrical high risk prethreshold ROP were included in this study. One eye of each baby was randomized to get either standard spot sized conventional pulsed mode diode laser or continuous mode LS TTT. There was no significant difference between structural or functional outcome in either group. The mean time taken for conventional diode laser was 20.07 minutes, while that for LS TTT was 12.3 minutes. LS TTT was 40% more time efficient than the conventional laser. It may be better suited for the very small fragile premature infants as it is quicker than the conventional laser.

Retinopathy of prematurity (ROP) remains one of the most important causes of childhood blindness.[1] In the 1980s, cryotherapy was the treatment of choice for the treatment of ROP.[2] However, with the advent of laser via indirect ophthalmoscopy delivery system, photocoagulation became the treatment of choice. Diode laser photocoagulation has proven to be safe and effective in the management of ROP.[3] Moreover, the systemic and local complications are much lesser with laser than with cryo.

The effectiveness of diode laser photocoagulation has been compared between confluent and non-confluent patterns,[4] transpupillary and transscleral approaches,[5] and pulsed mode versus near-continuous mode delivery.[6] However, there has been no study comparing between standard spot sized conventional pulsed mode diode laser and continuous mode large spot transpupillary thermotherapy (LS TTT).

Transpupillary thermotherapy (TTT) has been used earlier in the treatment of age-related macular degeneration,[7] choroidal melanomas[8] and retinoblastoma.[9] We report our experience in using continuous mode LS TTT for treatment of high risk prethreshold ROP.

## Materials and Methods

**Figure 1:**
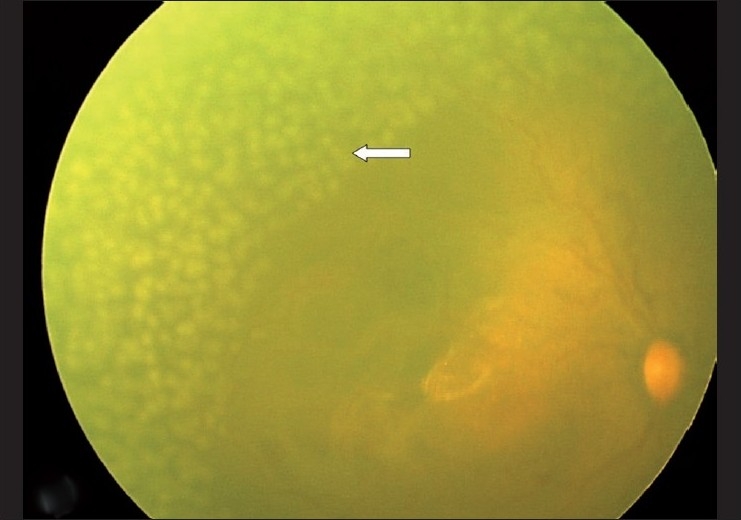
Retcam photo of right eye showing APROP with near confluent configuration application of laser using the conventional pulsed standard spot sized laser (white arrow)

**Figure 2:**
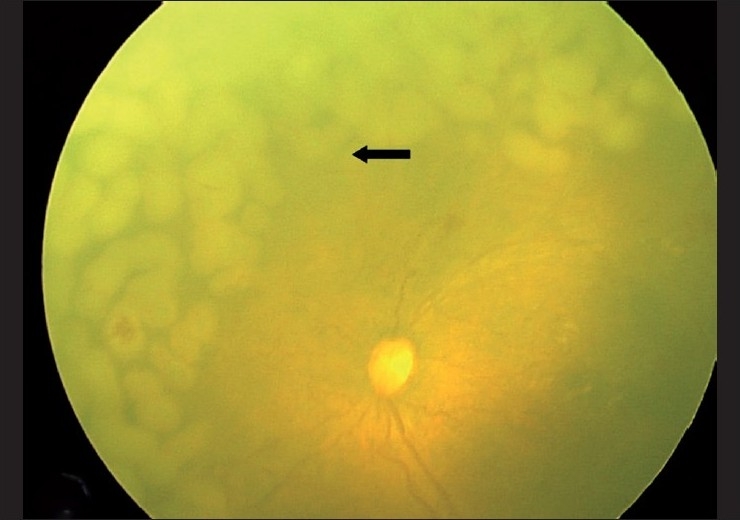
Retcam photo of left eye showing APROP with near confluent configuration application of laser using the continuous mode LS TTT (black arrow)

Five babies with bilateral symmetrical high risk prethreshold ROP were selected for this study. Two were with aggressive posterior retinopathy of prematurity (APROP), while the remaining three had high risk prethreshold ROP in zone 2. Babies with asymmetrical ROP were excluded. Informed consent was taken from the parents. International classification of ROP revisited was followed for classification, and early treatment for retinopathy of prematurity (ETROP) study guidelines were followed for treatment. One eye of each infant was randomized using a random number generator for treatment with either standard spot sized conventional pulsed mode laser or continuous mode LS TTT laser. The laser used in the study was infra-red diode (Oculight SLx, Iridex Co., CA, USA). All lasers were given under topical anesthesia with an anesthesiologist standby, by a single surgeon (PKS) using 28 diopter (D) lens. Entire avascular retina was ablated. The conventional laser with standard spot size was given with 150 ms pulsed mode, while the continuous mode group was given long pulse TTT with a large spot indirect. The spot size for the former was 360 μm and for the latter was 1.3 mm, with a 20 D lens. As we used a 28 D lens, the spot sizes for both were even bigger. We aimed for a near confluent configuration with the conventional laser indirect with gray white intensity burns separated by one-half spot size [Fig. 1]. For the LS TTT, as the laser was delivered in continuous mode, in order to create separate spots, the treating surgeon moved the laser beam to adjacent areas of retina at an appropriate speed so that areas of interruption between laser burns would occur [Fig. 2]. Photo documentation of all cases was done before and after laser and on each follow-up visit using Retcam[10](Clarity Medical Systems, Inc., Pleasanton, CA, USA). Immediate post laser picture and pattern of regression was similar in both the groups. All the babies were followed up for at least 1 year post laser. The laser-related complications compared between the two groups were apnea, bradycardia, corneal haze, iris burns, cataract, vitreous hemorrhage and hypotony. Total time taken for laser treatment and total energy used were also compared. Unfavorable outcome was defined as disease progressing to retinal detachment or severe macular drag. Functional outcome included visual behavior, refractive status and presence of amblyopia or strabismus at the end of 1 year after laser treatment. The similarities and differences in baseline characteristics, treatment and outcomes from this pilot study are described.

## Results

Totally, 10 eyes of five babies underwent laser photocoagulation with either the conventional pulsed mode standard spot size laser or the continuous mode LS TTT. Baseline characteristics of the enrolled five babies are given in Table 1 and the treatment parameters between the two groups are given in Table 2. Differences in the outcomes and details of treatment are presented in Table 3. There was no laser related complication in either group. All the babies were followed up for at least 1 year post laser. The structural outcome was good in both the groups. Visual outcome (tested in each eye separately) of both the groups was good as measured by the ability to pick up cake decorations. All the eyes had complete regression after laser and none ended up having unfavorable outcome.

**Table 1:** Baseline characteristics of enrolled babies

Characteristic	Enrolled babies (n = 5)
Gender	2 females, 3 males
Gestational age	Mean: 30.4 weeks (SD: 2.30, 95% CI: 27.5–33.3, median: 30.5, range: 28–33)
Birth weight	Mean: 1316 g (SD: 428, 95% CI: 784.4–1847.6, median: 1320, range: 800–1840)
Maternal risk factors	Nil
Neonatal risk factors	
Respiratory distress syndrome	All five
Oxygen	Mean: 14 days (95% CI: 7.03–20.97
supplementation	days, median: 13.5, range: 7–20)
Other complications	PT (1), sepsis (1), sepsis with IVH (1)
Status at time of receiving laser treatment	
Plus disease	All five
Zone	Zone 1 (2), Zone 2 (3)
Stage	Stage 3 (3), APROP (2)
Postnatal age	Mean: 5.52 weeks (95% CI: 2.4–9, median: 7, range: 4–10)
Post-conceptual age	Mean: 35.92 weeks (95% CI: 32.99– 38.85, median: 35, range: 32–38)

SD: Standard deviation, CI: Confidence interval, PT: Phototherapy, IVH: Intraventricular hemorrhage, APROP: Aggressive posterior retinopathy of prematurity

**Table 2:** Treatment parameters between the two groups

Treatment parameters	Standard spot sized conventional pulsed mode diode laser (n = 5)	Continuous mode LS TTT (n = 5)
Eye treated	Left (1), right (4)	Left (4), right (1)
Power	Mean: 292 mV (SD: 52.63, 95% CI: 226.65–357.35, median: 300, range: 270–330)	Mean: 300 mV (SD: 21.21, 95% CI: 273.66–326.34, median: 300, range: 230–370)
Duration of laser treatment	Mean: 20.72 minutes (SD: 13.05, 95% CI: 4.53– 36.93, median: 13.87, range: 2.46–25.29)	Mean: 12.32 minutes (SD: 9.24, 95% CI: 0.9–23.8, median: 21.24, range: 5.04–37.44)
Number of laser sittings	One (4), Two (1)	One (5)

SD: Standard deviation, CI: Confidence interval

**Table 3:** Outcomes of the two treatment modalities

	ROP treatment	
	Standard spot sized conventional pulsed mode diode laser (n = 5)	Continuous mode LS TTT (n = 5)
Immediate complications	Nil	Minimal bleed at ridge (1)
Sequelae	Nil (4), pale disk with nystagmus (1)	Nil (3), mild disk drag (1), pale disk with nystagmus (1)
Follow-up in months	Mean: 14.06 (SD: 3.09, 95% CI: 10.2–17.9, median: 15.75, range: 12–19.5)	
Vision	Perception of light (1), able to pick up cake decorations (4)	Perception of light (1), able to pick up cake decorations (4)
Objective refractive correction	–3 DS (1), nil (4)	3.5 DS × 180° (1), –4.5 DS (1), nil (3)

ROP: Retinopathy of prematurity, SD: Standard deviation, CI: Confidence interval, DS: Diopter sphere

## Discussion

Laser photocoagulation has become the standard treatment for ROP. Compared to cryotherapy, it has less systemic and local complications and thus better tolerated. However, conventional pulsed laser takes longer time duration especially in cases of APROP, where extensive avascular retina has to be covered. This can be stressful for the baby as there are higher chances of apnea and bradycardia due to prolonged scleral indentation. Continuous mode delivery was significantly more time efficient than pulsed mode delivery. Treatment time was reduced by almost 40%. This decrease in time required for laser could translate into less fatigue for the infant and could be clinically significant in fragile premature infants. Similar results are reported by Paysee *et al*.[6] However, they used near-continuous mode laser with standard spot size compared to continuous mode with a large spot laser as in the present study. There was no difference between the mean powers used between the two groups. In spite of the LS TTT being quicker and more efficient than the conventional standard spot sized laser, we found no difference in the complications rates between the two treatment modalities. Even though LS TTT does not cause any complications, the authors feel that the potential complications of large spot continuous laser would be over-treatment, difficulty in using large spot through small pupil and difficulty in covering small areas near the ridge and at the periphery.

In conclusion, our study demonstrates that continuous mode large spot LS TTT was significantly more time efficient compared to the standard size pulsed mode laser for treatment of high risk prethreshold ROP. This could be important while treating very small fragile preterm babies especially with APROP, mainly because we find no difference in the structural and functional outcomes between the two at the end of 1 year. The safety, efficacy and long-term outcomes of continuous mode LS TTT as compared with standard size pulsed mode laser for treatment of high risk pre-threshold ROP can, however, be established only through well-designed randomized controlled trials.

## References

[1] Steinkuller PG, Du L, Gilbert C, Foster A, Collins ML, Coats DK (1999). Childhood blindness. J AAPOS.

[2] Mousel DK, Hoyt CS (1980). Cryotherapy for retinopathy of prematurity. Ophthalmology.

[3] White JE, Repka MX (1997). Randomized comparison of diode laser photocoagulation versus cryotherapy for threshold retinopathy of prematurity: 3-year outcome. J Pediatr Ophthalmol Strabismus.

[4] Banach MJ, Ferrone PJ, Trese MT (2000). A comparison of dense versus less dense diode laser photocoagulation patterns for threshold retinopathy of prematurity. Ophthalmology.

[5] Seiberth V, Linderkamp O, Vardarli I (1997). Transscleral vs transpupillary diode laser photocoagulation for the treatment of threshold retinopathy of prematurity. Arch Ophthalmol.

[6] Paysse EA, Hussein MA, Miller AM, McCreery BK, Coats DK (2007). Pulsed mode versus near-continuous mode delivery of diode laser photocoagulation for high-risk retinopathy of prematurity. J AAPOS.

[7] Nagpal M, Nagpal K, Sharma S, Puri J, Nagpal PN (2003). Transpupillary thermotherapy for treatment of choroidal neovascularization secondary to age-related macular degeneration in Indian eyes. Indian J Ophthalmol.

[8] Oosterhuis JA, Journée-de Korver HG, Kakebeeke-Kemme HM, Bleeker JC (1995). Transpupillary thermotherapy in choroidal melanomas. Arch Ophthalmol.

[9] Abramson DH, Schefler AC (2004). Transpupillary thermotherapy as initial treatment for small intraocular retinoblastoma: Technique and predictors of success. Ophthalmology.

[10] Shah PK, Narendran V, Saravanan VR, Raghuram A, Chattopadhyay A, Kashyap M (2006). Screening for retinopathy of prematurity: A comparison between binocular indirect ophthalmoscopy and RetCam 120. Indian J Ophthalmol.

